# The formation pathway of flavor compounds in steamed Antarctic krill (*Euphausia superba*) based on untargeted metabolomics

**DOI:** 10.1016/j.fochx.2024.102075

**Published:** 2024-12-09

**Authors:** Tingting Yang, Yang Liu, Jing Bai, Yan Fan, Ye Chen, Ping Dong, Xia Yang, Hu Hou

**Affiliations:** a State Key Laboratory of Marine Food Processing & Safety Control, College of Food Science and Engineering, Ocean University of China, No.1299, Sansha Road, Qingdao, Shandong Province 266404, PR China; b Laboratory for Marine Drugs and Bioproducts, Qingdao Marine Science and Technology Center, No.168, Wenhai Middle Road, Qingdao, Shandong Province 266237, PR China; c Qingdao Institute of Marine Bioresources for Nutrition & Health Innovation, No.106, Xiangyang Road, Qingdao, Shandong Province 266200, PR China; d Sanya Oceanographic Institution, Ocean University of China, Sanya, Hainan Province 572024, PR China

**Keywords:** Steaming, Antarctic krill, Flavor compounds, Formation pathway, Untargeted metabolomics

## Abstract

This study investigated the impact of steaming on the flavor and metabolic profile of Antarctic krill, aiming to elucidate the pathways responsible for flavor development and metabolic shifts during processing. HS-SPME-GC–MS identified key volatile compounds, including alcohols, aldehydes, ketones and so on. The results demonstrated a significant increase in nonanal content from 2.23 ± 0.06 μg/kg to 8.14 ± 1.26 μg/kg after steaming. The formation pathways of two key flavor compounds, nonanal and 1-octen-3-ol, were attributed to fatty acid degradation. Hierarchical clustering and volcano plot showed metabolic shifts between raw and steamed krill, with differential metabolites like hydroquinone and gamma-aminobutyric acid emerging as key contributors to flavor changes. Furthermore, metabolic network further linked these shifts to reactions involving amino acids, nucleotide and other compounds during steaming, impacting the overall taste.

## Highlights

• E-2-octenal, nonanal, 1-octen-3-ol are the key flavor compounds in steamed krill.
• Steaming induces the formation of nonanal, 1-octen-3-ol via fatty acid degradation.
• Lipid degradation and amino acid reactions contribute to the development of flavor.

## Graphical abstract

**Figure:**
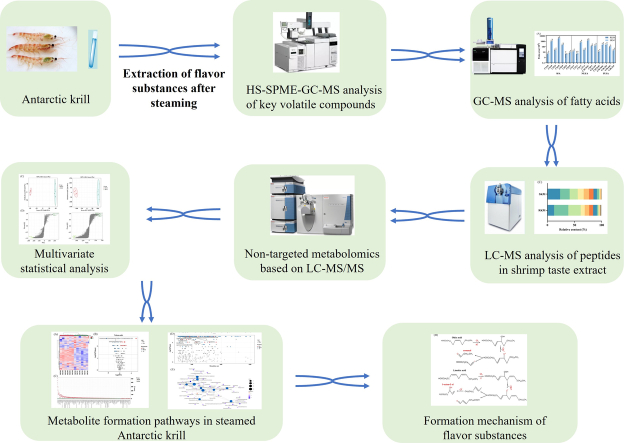

## 1 Introduction

Antarctic krill (*Euphausia superba*) has the advantages of large biomass, rich nutrition, high protein and low fat (Hamner & Hamner, 2000), and it is abundant in essential amino acids, as well as DHA and EPA, along with other bioactive compounds (Jiang et al., 2024). In the waters off the western Antarctic Peninsula, krill make up 75–90 % of the total biomass (McBride et al., 2021), with estimates suggesting that the krill reserves in this region could reach billions of tonnes. Antarctic krill has broad application prospects and is considered a potential future food source. Currently, Antarctic krill products primarily include krill powder, dried krill, and krill oil, which are produced through steaming processes (Trathan et al., 2021). However, the use of steam in heat processing has significant impact on the volatile compounds in meat (Khan et al., 2015).

The characteristic flavor of Antarctic krill meat was developed through chemical reactions such as protein hydrolysis and fat oxidation (Li et al., 2022). Similarly, Zhang, Cao, et al. (2019) identified and quantified the characteristic flavor compounds in golden pomfret fillets, analyzing the generation of volatile flavor compounds from the perspectives of Strecker degradation, protein hydrolysis, and lipid oxidative degradation. In another study, Mall and Schieberle (2016) used aroma extract dilution analysis to identify the primary aroma compounds in raw and cooked shrimp, revealing that different heat treatments caused variations in the types and quantities of aroma compounds in shrimp meat. While the qualitative analysis of flavor substances and the quantitative analysis of key volatile compounds in food have become well-established, there is a lack of research into the interactions between these flavor compounds and their formation mechanisms.

The flavor is a key factor that influences people's preference for food. Previous studies have investigated the volatile flavor compounds and the impact of heat treatment on the flavor profile of Antarctic krill. Fan et al. (2017) used HS-SPME and GC–MS to analyze the volatile flavor compounds in Antarctic krill, detecting a total of 42 volatile substances responsible for its unique flavor. Of these, seven compounds made significant contributions to the flavor profile of Antarctic krill. Bai et al. (2022) conducted sensory evaluations along with electronic tongue and nose analyses, as well as GC–MS, to compare the flavor of Antarctic krill and white shrimp, showing notable changes in the characteristic flavor compounds after steaming and boiling. Additionally, Zhang et al. (2018) investigated the effects of different heating methods on the content of six non-volatile reactive compounds (NRCs) in Antarctic krill and Pacific white shrimp, demonstrating that adjusting heating conditions effectively controlled the NRC levels.

However, researchers have primarily focused on the qualitative and quantitative assessments of individual flavor compounds when studying the flavor of steamed aquatic products, lacking a comprehensive characterization of the multiple factors influencing flavor (Zhang, Ma, & Dai, 2019). Additionally, research on the interactions between flavor components and the formation mechanisms of key compounds remains limited (Liang et al., 2022). Currently, the effects of steaming on the flavor quality of Antarctic krill are not well understood, and the mechanisms of flavor formation require further investigation. Therefore, it is essential to explore the processes and mechanisms responsible for the characteristic flavor of steamed Antarctic krill. The present study aims to elucidate the formation pathway of key flavor compounds during processing. The untargeted metabolomics approach was employed to analyze the alterations of typical flavor compounds in Antarctic krill pre- and post- steaming, thereby establishing a foundation for quality control measures pertaining to Antarctic krill. These results will establish a theoretical foundation for comprehending the metabolic alterations and formation pathways of flavor compounds.

## 2 Materials and methods

### 2.1 Materials and reagents

Antarctic krill was supplied with the China National Aquatic Products Corporation (Zhoushan, Zhejiang, China). All reagents are analytical grade.

### 2.2 Preparation of taste extracts from Antarctic krill

The thawed Antarctic krill meat was homogenized with ultrapure water at a ratio of 1:10 (w/v) and then centrifuged at 9000 rpm for 20 min at 4 °C. The resulting supernatant of raw Antarctic krill meat (RKM) was collected as the taste extract. For the steamed samples, raw krill was steamed for 5 min. The preparation of the natural flavor extract from steamed krill meat (SKM) followed the same procedure as for the raw krill meat.

### 2.3 GC–MS analysis of volatile flavor compounds

The volatile compounds of Antarctic krill meat were extracted using HS-SPME (Fan et al., 2017). The minced SKM or RKM samples (3.0 g), along with 3 mL saturated NaCl solution and 2, 4, 6-trimethylpridine were introduced into a 20 mL headspace bottle, respectively. An SPME fiber (65 μm, PDMS/DVB, Supelco, Bellefonte, PA, USA) was inserted into the headspace of the vial and extracted for 45 min at 65 °C. The fiber was transferred to an injection port and desorbed for 5 min at 250 °C.

The volatile flavor compounds in SKM or RKM samples were identified using GC–MS (7000 D, Agilent Technologies Inc., USA) with an HP-5MS capillary column (30 m × 0.32 mm × 0.25 μm). The heating program was as follows: the initial oven temperature was set at 40 °C for 3 min, then increased to 250 °C at a rate of 5 °C/min, and held at 250 °C for 5 min. The sample was introduced into the GC at a flow rate of 1.0 mL/min using helium (99.999 %), with an injector temperature of 250 °C. The detector temperature was set at 250 °C, with a mass range of 35–350 *m*/*z*.

The identification of volatile flavor compounds by GC–MS was performed by searching the NIST20 mass spectrometry database. N-Alkanes (C8-C30) were employed to calculate linear retention indices of volatile compounds. The content of volatile compounds was calculated according to the method of Kaseleht et al. (2011). In brief, each sample was spiked with 0.0465 μg of the internal standard 2,4,6-trimethylpyridine, and the compounds were relatively quantified based on the content and peak area of the internal standard.

### 2.4 Evaluation of odor activity values (OAV) of volatile compounds

OAV is employed to evaluate the contribution of a volatile flavor compound to the overall flavor profile of a sample. The OAV is calculated using the following formula:

OAV=C/OT

where OAV represents the odor activity value; C denotes the content of the volatile compound detected in the sample (μg/kg); OT refers to the literature-based odor threshold of this compound (μg/kg).

### 2.5 Fatty acids analysis using GC–MS

Antarctic krill meat (15 g) was combined with 200 mL of chloroform and 100 mL of methanol, then stirred thoroughly and placed at 4 °C overnight. The filtrate was collected and mixed with 0.9 % sodium chloride solution (5:1, v/v). After standing for 12 h, the lower organic phase was dried. The dried sample (20 g) were combined with 2 mL of 10 % concentrated sulfuric acid in methanol and reacted at 60 °C for 15 min. The cooled samples were then mixed with 2 mL of n-hexane, and the upper solution was filtered through a non-polar 0.22 μm filter after standing for 10 min.

The fatty acid composition of raw and steamed krill meat was analyzed using GC–MS (7000 D, Agilent Technologies Inc., USA) with an HP-INNOWAX quartz capillary column (30 m × 0.32 mm × 0.25 μm) following the method of Valasi et al. (2021). The sample was introduced into the GC with helium at a flow rate of 1.0 mL/min. The heating program was as follows: the initial oven temperature was set at 120 °C, then increased to 210 °C at a rate of 5 °C/min and maintained at 210 °C for 10 min. The mass spectrometer operated in electron ionization mode with a mass scan range of 50 to 500 *m*/*z* in full-scan mode. The electron energy was set at 70 eV, with the ion source temperature at 230 °C and the quadrupole temperature at 250 °C.

### 2.6 Analysis of peptides in krill taste extract

The Antarctic krill meat (6 g) was homogenized with a solid-liquid ratio of 1:4, and then centrifuged at 9000 rpm for 30 min at 4 °C. The resulting supernatant was mixed with acetonitrile to precipitate macromolecular proteins. After centrifugation, the supernatant was obtained and then freeze-dried. The resulting powder was dissolved (10 mg/mL), desalted, and then analyzed by an ultra-performance liquid chromatography (Dionex UltiMate 3000, Thermo Fisher Scientific Inc., Waltham, USA) coupled with a quadrupole Orbitrap mass spectrometer (Q Exactive, Thermo Fisher, USA).

The separation conditions for UHPLC were as follows: Mobile phase A consisted of 0.1 % formic acid in water, while mobile phase B was 0.1 % formic acid in acetonitrile. The analysis was performed using an Agilent Advanced Bio Peptide Map C18 column (150 mm × 2.1 mm, 2.7 μm particle size). The flow rate was set at 0.25 mL/min with an injection volume of 30 μL. The total run time was 46 min, and the column temperature was maintained at 40 °C.

The mass spectrometer operated in electrospray positive ion (ESI+) mode with an electrospray voltage of 5500 V. The scanning range for mass spectrometry was 300–1500 *m*/*z*. The atomization gas pressure was set to 60 psi, the auxiliary heating air pressure to 50 psi, the air curtain pressure to 35 psi, and the ion source temperature was kept at 525 °C. The flavor peptides and amino acids were assessed based on the BIOPEP database (https://biochemia.uwm.edu.pl/biopep-uwm/), followed by the prediction of their flavor characteristics.

### 2.7 Analysis of amino acid cleavage sites of peptide chain in krill taste extract

A 0.2 g portion of the lyophilized sample underwent derivatization according to the method of Shi et al. (2013). The derivatized sample was then vacuum-dried and dissolved. The solution was incubated in a water bath at 54 °C for 45 min and then vacuum-dried. The dried sample was dissolved in 80 % ethanol and extracted with n-hexane. The lower phase was filtered through a 0.22 μm filter membrane.

Free amino acids (FAA) were analyzed using HPLC with an Agilent 1260 Infinity system (Agilent Technologies Co. Ltd., California, USA), equipped with a UV-detector. The analysis was conducted using a ZORBAX Eclipse XDB-C18 column (4.6 × 250 mm × 5 μm) at 254 nm. Mobile phase A consisted of 0.1 mol/L sodium acetate-acetonitrile (97:3, v/v, pH 6.5), while mobile phase B was 80 % acetonitrile in water. The samples were eluted at a flow rate of 0.8 mL/min with the following gradient: 0–15 min, 95–84 % A; 15–35 min, 84–50 % A; 35–38 min, 50–95 % A; 38–40 min, 95 % A.

### 2.8 The approach of non-targeted metabolomics based on LC-MS/MS

#### 2.8.1 Liquid chromatography conditions

The liquid chromatography analysis was performed using a Vanquish UHPLC System (Thermo Fisher Scientific, USA) with a method slightly modified from Zelena et al. (2009). Chromatography was conducted using an ACQUITY UPLC® HSS T3 column (150 × 2.1 mm, 1.8 μm) (Waters, Milford, MA, USA), with the column temperature set at 40 °C.

The Antarctic krill sample was mixed with 0.75 mL of methanol-chloroform solution (9:1, v/v) and 0.25 mL of water. The mixture underwent ball milling homogenization at 50 Hz for 120 s, followed by ultrasonication for 30 min and ice bath treatment for an additional 30 min. The homogenate was then centrifuged at 12,000 rpm at 4 °C for 10 min. The resulting supernatant was collected and concentrated to dryness. Subsequently, 200 μL of 2-chloro-L-phenylalanine solution (4 ppm in 50 % acetonitrile) was added accurately to the dried residue. The mixture was filtered and prepared for LC-MS analysis.

#### 2.8.2 Mass spectrum conditions

Mass spectrometric detection of differential metabolites was done using an Orbitrap Exploris 120 (Thermo Fisher Scientific, USA) with an ESI ion source according to the method of Want et al. (2013). Detection was conducted in Full MS-ddMS2 mode (data-dependent MS/MS).

#### 2.8.3 Data analysis

It was employed for multivariate data analysis and model construction (Thévenot et al., 2015). After scaling the data, orthogonal-partial least-square discriminant analysis (OPLS-DA) was used to build the models. Metabolic profiles were visualized using score plots, where each point represented a sample. Corresponding S-plots were generated to highlight the metabolites that influenced the clustering of the samples. The *P*-value, VIP from OPLS-DA, and fold change (FC) were applied to identify variables contributing to classification. Finally, metabolites with P-value <0.05 and VIP > 1 were considered statistically significant.

#### 2.8.4 Pathway analysis

Differential metabolites were subjected to pathway enrichment analysis using MetaboAnalyst, which combines both pathway enrichment and topology analyses (Xia & Wishart, 2011).

### 2.9 Statistical analysis

All experiments were performed at least three times and the data were expressed as the mean value ± standard deviation. Statistical analysis was performed using SPSS 19.0 software.

## 3 Results and discussion

### 3.1 Effects of steaming on the relative content of volatile flavor compounds in Antarctic krill

The exquisite flavor of krill meat primarily depends on the composition of volatile compounds. In this study, HS-SPME combined with GC–MS was used to analyze the types and concentrations of volatile flavor compounds in steamed krill. The RKM and SKM samples were detected to contain a total of 39 volatile flavor compounds, including alcohols, aldehydes, esters, and so on (Table 1). The species and concentrations of these volatile compounds differed between SKM and RKM. Alcohols and aldehydes were formed through lipid degradation, the Maillard reaction, Strecker degradation, and the interaction of their metabolites (Al-Dalali et al., 2022).

**Table 1:** Analysis of volatile compounds in Antarctic krill.

Classification	Compounds	RI	Identifiction	Content (μg/kg)		Odor description
				RKM	SKM	
*Alcohols*						
1	2,6-nonadiene-1-ol	–	MS	N	3.03 ± 0.23	green, vegetable
2	1-octene-3-ol	978	MS, RI	N	0.49 ± 0.04	mushroom, green
3	2-ethyl-1-hexanol	1029	MS, RI	N	14.68 ± 4.12	mushroom
4	2,6-dimethyl cyclohexanol	1108	MS, RI	N	1.15 ± 0.03	flower
5	α-terpineol	1192	MS, RI	N	0.40 ± 0.10	flower
6	5-nondecene-1-ol	1206	MS, RI	N	0.80 ± 0.12	
7	phytol	2114	MS, RI	N	1.25 ± 0.53	flower
*Aldehydes*						
1	E-2-octenal	972	MS, RI	6.09 ± 0.97	3.53 ± 0.21	nutty
2	phenylacetaldehyde	1043	MS, RI	N	2.18 ± 0.65	green, honey
3	nonanal	1104	MS, RI	2.23 ± 0.06	8.14 ± 1.26	fat, citrus, green
4	2,4-dimethyl benzaldehyde	1178	MS, RI	0.44 ± 0.09	N	almond
5	hexadecaldehyde	1817	MS, RI	N	1.25 ± 0.33	fat
*Ketones*						
1	1-phenyl-2-pentanone	–	MS	0.20 ± 0.01	N	
2	6-methyl-5-heptene-2 -one	986	MS, RI	4.54 ± 0.43	2.28 ± 0.43	fruity
3	3-ethyl-4-heptanone	1048	MS, RI	N	0.35 ± 0.09	
4	heptanophenone	1065	MS, RI	N	0.88 ± 0.02	fruity
5	acetophenone	1066	MS, RI	N	0.89 ± 0.01	sweet, fruity
6	2,5-hexanedione	1244	MS, RI	N	0.29 ± 0.07	smelly
7	benzophenone	1633	MS, RI	N	0.22 ± 0.03	rose, sweet
*Esters*						
1	2-methyl-butanoic acid methylester	–	MS	1.09 ± 0.08	N	fruity
2	methyl acetate	1173	MS, RI	N	0.54 ± 0.09	aroma
3	butyl benzoate	1376	MS, RI	N	0.28 ± 0.03	fruity
4	dibutyl phthalate	1883	MS, RI	3.50 ± 0.30	N	
*Hydrocarbons*						
1	ethyl benzene	853	MS, RI	26.33 ± 0.48	1.08 ± 0.10	aromatic
2	α-pinene	930	MS, RI	13.17 ± 0.39	N	citrus
3	γ-terpinene	1008	MS, RI	6.91 ± 0.21	N	rosin，resin
4	D-limonene	1028	MS, RI	17.45 ± 1.08	N	orange, lemon
5	undecane	1099	MS, RI	0.72 ± 0.06	N	alkane odor
6	dodecane	1101	MS, RI	1.00 ± 0.08	N	alkane odor
7	2-methyl-naphthalene	1295	MS, RI	N	2.56 ± 0.35	naphthalene odor
*Others*						
1	dimethyl disulfide	–	MS	0.36 ± 0.04	N	onion, cabbage
2	methoxy-phenyl oxime	905	MS, RI	14.27 ± 0.02	18.79 ± 0.59	
3	2-methyl-benzofuran	1199	MS, RI	3.26 ± 0.30	N	
4	benzothiazole	989	MS, RI	N	2.52 ± 0.26	caramel
5	2,4-di-tert-butylphenol	1225	MS, RI	N	0.23 ± 0.05	potion
6	butyl hydroxytoluene	1513	MS, RI	0.38 ± 0.01	0.69 ± 0.05	
7	tetradecanoic acid	1517	MS, RI	0.51 ± 0.09	2.17 ± 0.17	
8	2-pentadecyl furan	1865	MS, RI	N	0.20 ± 0.01	
9	N-hexadecanoic acid	1961	MS, RI	1.89 ± 0.06	2.50 ± 0.11	

Note: - indicates not calculated; N means not detected; Odor description refers to literature.

The alcohols produced by steaming in this study predominantly exhibited green, floral, and vegetal notes, which may significantly influence the flavor profile of Antarctic krill meat (Lorenzo et al., 2013). A notable change in alcohol concentrations was observed after steaming. As shown in Table 1, seven alcohols were detected in Antarctic krill post-steaming, most of which contributed green, vegetal, and floral flavors (Xu et al., 2021). Among these, 2,6-nonadien-1-ol imparted a green and vegetal fragrance, while α-terpineol provided a floral aroma (Xu et al., 2021). 1-Octen-3-ol, with the higher odor activity value (OAV) in Antarctic krill, was characterized by a mushroom and green aroma and is also considered a key contributor to the earthy flavors found in aquatic products. Five aldehydes were detected in Antarctic krill, including phenylacetaldehyde and hexadecanal, which were found in steamed Antarctic krill, with a significant increase in nonanal content. Phenylacetaldehyde imparted green and honey-like aromas, while acetaldehyde contributed a fatty note, both potentially playing a key role in the flavor profile of Antarctic krill meat (Serot et al., 2001). The content of nonanal in Antarctic krill increased from 2.23 to 8.14 μg/kg after steaming, contributing a fatty aroma. This increase is believed to result from the thermal oxidation or degradation of unsaturated fatty acids, particularly linoleic or linolenic acids (An et al., 2020; Cao et al., 2014).

Ketones are typically produced through the oxidation of lipids and the Strecker degradation of amino acids (Zhang et al., 2020). In total, seven ketone compounds were detected in Antarctic krill, contributing to the fruity aroma of krill products (Sarnoski et al., 2010). Ester compounds, which are esterification products of carboxylic acids and alcohols formed during lipid metabolism, play a key role in determining the flavor characteristics of Antarctic krill (Carrapiso et al., 2002). Steaming led to the formation of methyl acetate and butyl benzoate, both with fruity aromas, as well as benzothiazole, which imparted a caramel-like flavor.

The OAV is an important indicator for assessing the overall contribution of a single compound to the odor profile of Antarctic krill meat (Zhang, Ma, et al., 2019). The impact of volatile compounds on the sample's flavor depends not only on their concentration but also on their odor threshold value (Zhu et al., 2016). Generally, compounds with an OAV greater than 1 are typically regarded as active and make a significant contribution to the overall flavor of the sample (Hu et al., 2021). As shown in Table 2, among the aldehyde compounds, the content of E-2-octenal decreased after steaming, but its OAV remained significantly greater than 1. The OAV of phenylacetaldehyde and nonanal in SKM were 2.18 and 8.14, respectively, indicating that aldehydes played a crucial role in the characteristic flavor of Antarctic krill. Overall, E-2-octenal, phenylacetaldehyde, and nonanal were identified as key contributors to the flavor of steamed Antarctic krill, imparting grassy, citrus, and floral notes to SKM (Zhao et al., 2020).

**Table 2:** Odor activity evaluation and odor description of volatile compounds in Antarctic krill.

Classification	Compounds	Threshold (μg/kg)^a^	OAV		Odor description^b^
			RKM	SKM	
*Alcohols*	1-octene-3-ol	1	N	0.49	mushroom, green
	α-terpineol	330	N	0	flower
*Aldehydes*	E-2-octenal	3	2.03	1.18	nutty
	phenylacetaldehyde	1	N	2.18	green, honey
	nonanal	1	2.23	8.14	fat, citrus, green
*Ketones*	6-methyl-5-heptene-2-one	50	0.09	0.05	fruity
*Hydrocarbon*	D-limonene	10	1.75	N	orange, lemon

Note: N means not detected; a threshold reference; b odor Description: refer to previous literature.

### 3.2 Effects of lipid oxidative degradation on flavor formation in steamed Antarctic krill

**Fig. 1:**
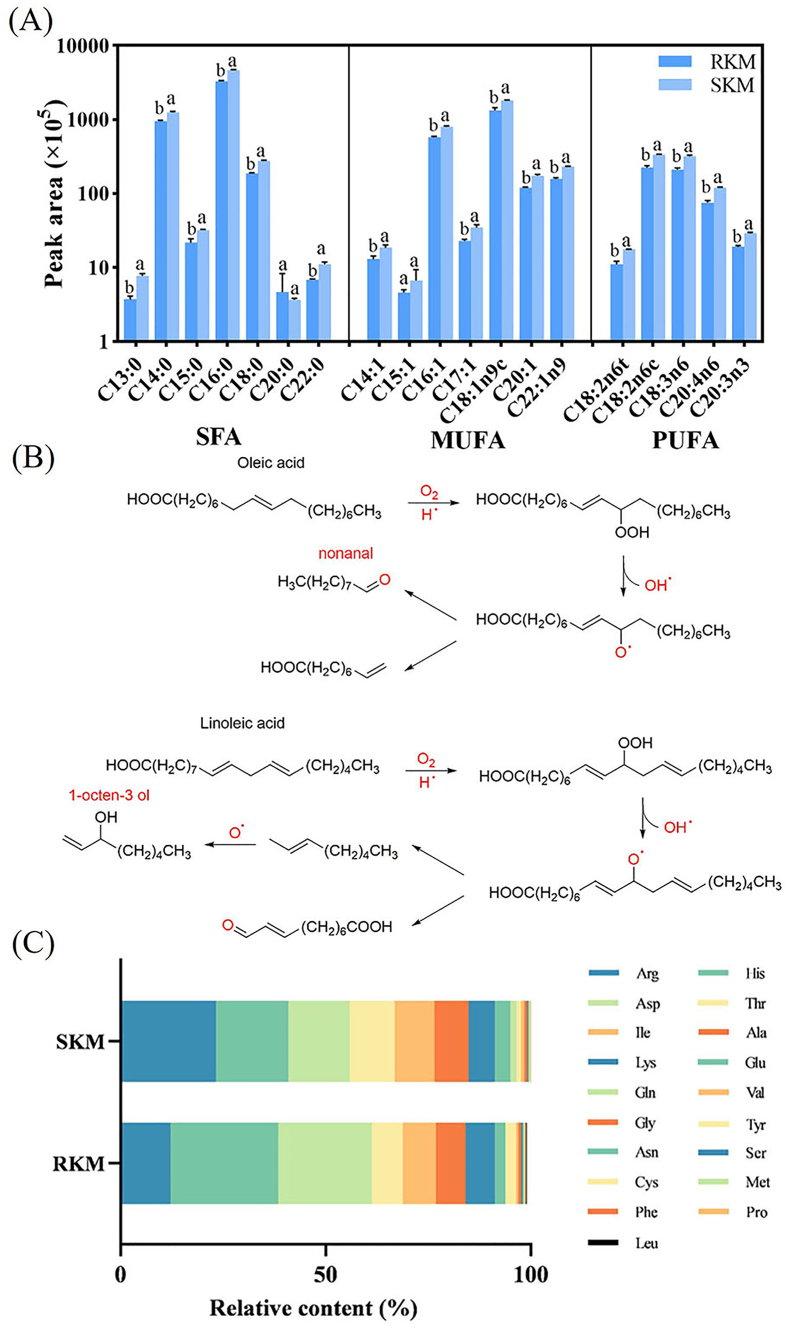
Effects of oxidative degradation of fat and proteolysis on flavor compounds. (A) Fatty acid composition of steamed Antarctic krill. Different letters showed significant differences between RKM and SKM for the same fatty acids (*P* < 0.05). (B) Possible pathways for the formation of nonanal and 1-octen-3-ol during the steamed in Antarctic krill. (C) Analysis of amino acid break sites of steamed Antarctic krill.

The lipids in Antarctic krill serve as essential precursors for the formation of volatile flavor compounds, which are subsequently converted into fatty acids during steaming (Matsui et al., 2003). The volatile compounds produced after steaming are mainly the result of lipid oxidation and the interactions between products of lipid oxidation and those of the Maillard reaction (Wang et al., 2022). A total of 19 fatty acids were identified in RKM and SKM samples (Fig. 1A). After steaming, the total contents of saturated fatty acids (SFA), monounsaturated fatty acids (MUFA), and polyunsaturated fatty acids (PUFA) increased significantly, indicating that the fats in Antarctic krill were degraded by the steaming process. Lipid oxidative degradation is a key pathway for flavor formation, and certain unsaturated fatty acids, such as oleic and linoleic acids, are particularly prone to oxidation into peroxides during steaming due to the presence of double bonds in their molecules. The further decomposition of these peroxides generates ketones, aldehydes, alcohols, and other compounds. Previous studies have shown that hydroperoxides can also form from SFA, MUFA, and esters (Abdelrazig et al., 2020).

The flavor components of Antarctic krill, including ketones, hydrocarbons, and aldehydes, are primarily generated through lipid degradation and oxidation during steaming (Matsui et al., 2003). Linear fatty aldehydes and alkanes are typical lipid oxidation products (Farvin et al., 2012). Nonanal is a product formed through the oxidation of oleic acid (Cao et al., 2014), while the odor-active compound 1-octen-3-ol, found in krill meat samples, is formed through the oxidative degradation of linoleic acid (Fig. 1B).

### 3.3 Analysis of polypeptides in flavor extract of steamed Antarctic krill

Polypeptides can exhibit taste characteristics such as bitterness, umami, sweetness, and sourness, playing a crucial role in enhancing food flavor (Warren et al., 2017). Many known umami peptides are derived from hydrolyzed animal proteins (Zelena et al., 2009). For instance, Want et al. (2013) identified umami peptides such as SEE, EEE, EDE, and DES from fish protein hydrolysate. As shown in Table 3, several peptide sequences were identified from taste extracts of heat-treated Antarctic krill.The peptides EDPIVP and EDPLVP had relatively higher sweetness and umami scores compared to others (Table 3). It has been reported that the taste characteristics of peptides may be related to the types of amino acid residues within their sequence, with peptides containing more umami amino acid residues tending to have a stronger umami taste (Horai et al., 2010).

**Table 3:** Cleavage sites and taste of peptides in heat-induced Antarctic krill.

Sequence	Cleavage sites	Confidence	Taste prediction (scores)				
			Sour	Sweet	Bitter	Salty	Umami
ETGLKYFPSQGNFIVIDFGIDGDEVFQYLL	**KE**TGLKYFPSQGNFIVIDFGIDGDEVFQYL**LS**	>95 %	0.2667	0.3000	0.9000	0.1333	0.3000
PAGMVVNPITSTQQGNLSTQQPSIIAH	**PP**AGMVVNPITSTQQGNLSTQQPSIIA**HN**	>95 %	0.0370	0.3333	0.3704	N	0.0370
LFDYPIFFALGLFSSTFGFIMLGAM	**VL**FDYPIFFALGLFSSTFGFIMLGA**MG**	>95 %	0.0400	0.2400	1.0000	0.0400	0.0400
DPSGDRLRQWLDIDKDIFYNPNK	**ND**PSGDRLRQWLDIDKDIFYNPN**KI**	>95 %	0.3043	0.2174	0.6957	0.2174	0.2609
DAERIHGISTELAMEQGVSLQEVL	**YD**AERIHGISTELAMEQGVSLQEV**LA**	>95 %	0.2917	0.2917	0.5833	0.0417	0.4167
AGFAGDDAPRAVFPSIVGRPR	**KA**GFAGDDAPRAVFPSIVGRP**RH**	>95 %	0.1429	0.5714	1.0000	0.1429	0.2381
SGISNKIASLLKQDWGSLE	**RS**GISNKIASLLKQDWGSL**EE**	>95 %	0.2105	0.2632	0.6316	0.0526	0.1053
KSVVKEVTSAGGDIVLFI	**LK**SVVKEVTSAGGDIVLF**ID**	>95 %	0.2778	0.6111	0.7778	0.0556	0.2222
DPSQSLSETEFVGL	**WD**PSQSLSETEFVG**LA**	>95 %	0.2857	0.2143	0.7143	0.0714	0.4286
ENVLITTPKTN	**QE**NVLITTPKT**NN**	>95 %	0.2727	0.2727	0.6364	N	0.0909
TAVHHPLIDIVA	**KT**AVHHPLIDIV**AD**	>95 %	0.0833	0.4167	0.7500	0.0833	0.0833
DQVLLSGVQGIT	**AD**QVLLSGVQGI**TD**	>95 %	0.0833	0.3333	0.6667	0.0833	0.0833
QDVIIKELQGS	**IQ**DVIIKELQG**SE**	>95 %	0.3636	0.2727	0.6364	0.0909	0.2727
PIDEPVIP	**QP**IDEPVI**PG**	>95 %	0.3750	0.5000	0.8750	0.2500	0.3750
ELPDPVIP	**RE**LPDPVI**PE**	>95 %	0.2500	0.5000	1.0000	0.1250	0.3750
EDPIVP	**EE**DPIV**PP**	>95 %	0.5000	0.5000	0.6667	0.3333	0.5000
EDPLVP	**SE**DPLV**PS**	>95 %	0.5000	0.5000	1.0000	0.3333	0.5000

Note: N means not detected; The BIOPEP database for peptides and amino acids (https://biochemia.uwm.edu.pl/biopep-uwm/).

The study revealed that the umami taste enhancement thresholds of ALPEEV and EAGIQ were 1.52 mmol/L and 1.94 mmol/L, respectively (Sud et al., 2007). These peptides, known to possess umami taste, were predicted and scored using the same method applied in this study. The results showed umami scores of 0.8333 for ALPEEV and 0.4000 for EAGIQ. In comparison, the umami scores of EDPIVP and EDPLVP, identified in this study, were both 0.5000, falling between the values of 0.4000 and 0.8333. This suggests that the umami threshold of EDPIVP and EDPLVP might range between 1.52 and 1.94 mmol/L, confirming the reliability of the prediction method used. Therefore, the polypeptides AGFAGDDAPRAVFPSIVGRPR, ELPDPVIP, EDPIVP, and EDPLVP, isolated from steamed Antarctic krill, exhibit both umami and sweetness. Additionally, these peptides showed relatively high bitterness scores, likely due to the presence of hydrophobic amino acids in their sequences.

### 3.4 Analysis of peptide chain amino acid cleavage site of steamed Antarctic krill

The principle of the Edman degradation method involves modifying the N-terminal of water-soluble small peptides in the sample and subsequently cleaving them. This allows the determination of the amino acid cleavage sites at the N-terminus of larger peptide chains during the formation of smaller peptide hydrolysis products, based on changes in free amino acid content. After steaming, a significant increase in the content of certain amino acids indicates the specific cleavage sites in the peptide chain. For instance, the contents of E, R, T, A, Y, and V increased significantly after steaming (Fig. 1C), suggesting that the peptide chain cleaved at these sites during the steaming process.

### 3.5 Non-targeted metabolomics analysis of Antarctic krill during steaming

#### 3.5.1 Multivariate statistical analysis

**Fig. 2:**
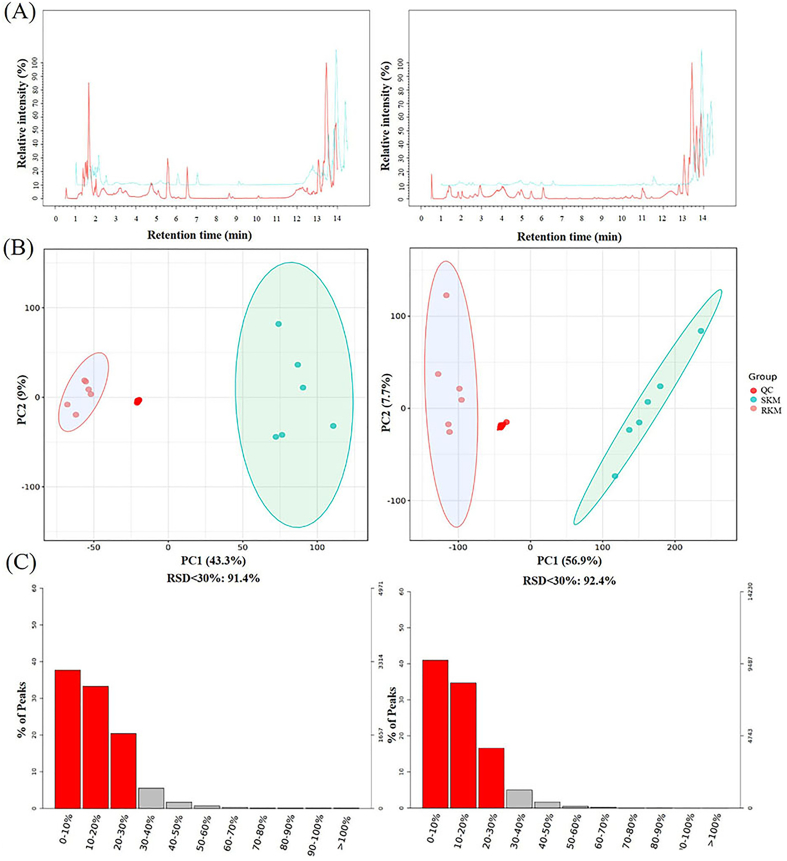
(A) Base peak chart of typical samples. (B) Principal component analysis score plot of quality control. (C) Relative standard deviation distribution of quality assurance.

**Fig. 3:**
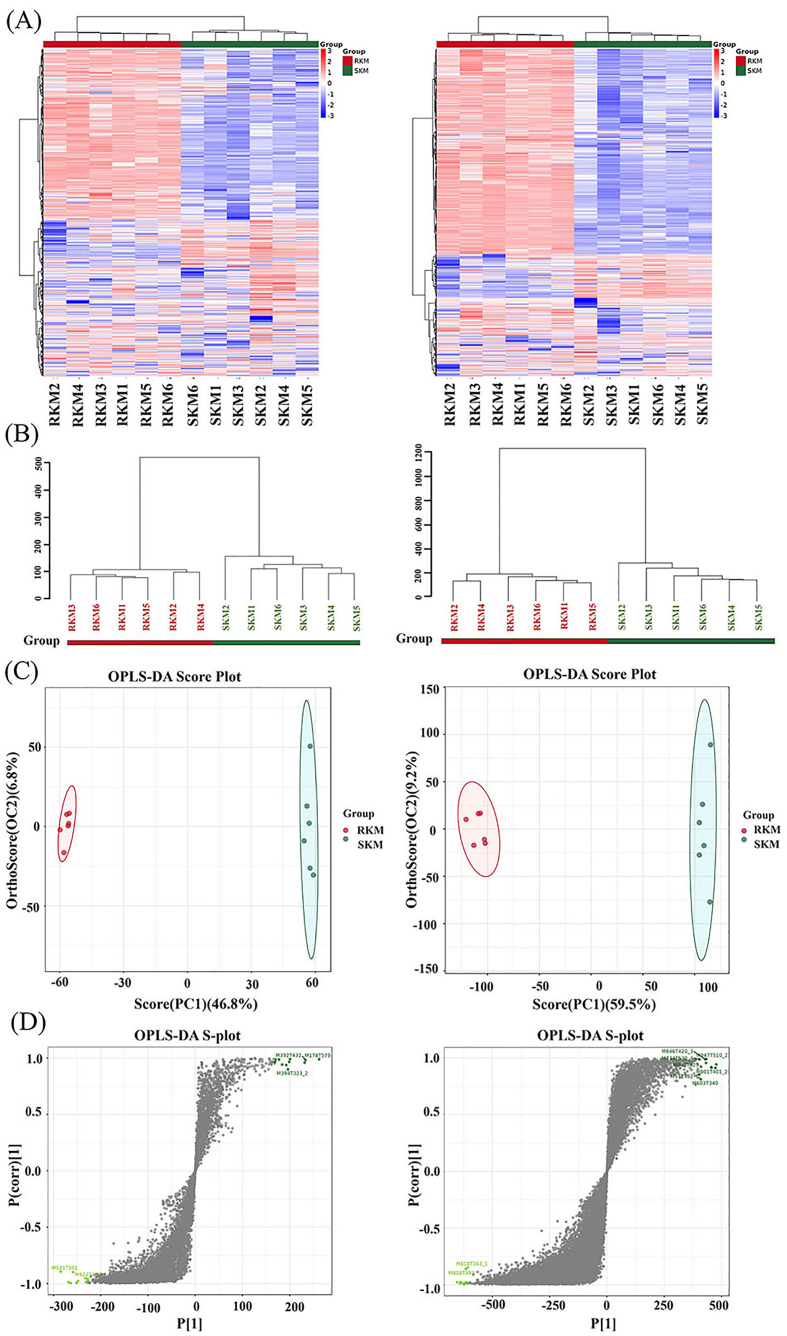
Multivariate statistical analysis. (A) Hierarchical clustering heat map of metabolites. (B) Clustering analysis. (C) OPLS-DA Score Plot. (D) OPLS-DA s-plot.

Non-targeted metabolomics was used to identify and analyze metabolites in Antarctic krill before and after steaming, using both positive (left) and negative (right) ion scanning modes. The base peak chromatogram (Fig. 2A) of the samples showed that the trends across different groups of Antarctic krill samples were similar, indicating good repeatability. Quality control (QC) procedures (Fig. 2B) were performed to remove characteristic peaks that did not meet the requirements (RSD > 30 %), resulting in a higher-quality dataset (Fig. 2C). The PCA score plot demonstrated that the QC samples clustered together, confirming the reliability and reproducibility of the analysis. Following LC-Orbitrap-MS analysis, a total of 7572 metabolites were detected. As shown in Fig. 3A, while the content of most metabolites decreased, some compounds increased in SKM. This indicates that steaming caused significant changes in the metabolite content of Antarctic krill. Fig. 3B showed the hierarchical clustering tree diagram of samples before and after steaming. The samples were classified into two distinct categories, indicating significant changes between SKM and RKM samples. OPLS-DA (Orthogonal Partial Least Squares Discriminant Analysis) is effective for filtering irrelevant information and accurately analyzing the differences and associations of metabolites across different samples (Heidrich et al., 2021). As shown in the scores plot (Fig. 3C), the separation between RKM and SKM reflects notable changes in the metabolites of Antarctic krill after steaming. The samples within the RKM group showed good aggregation, while some separation within the SKM group could be attributed to thermal induction. The OPLS-DA analysis yielded key parameters: R^2^X = 0.536, R^2^Y = 0.999, and Q^2^ = 0.964, all exceeding 0.5, indicating that the model is robust for identifying differential metabolites. The OPLS-DA S-plot (Fig. 3D) highlights the differential metabolite profiles crucial for distinguishing between RKM and SKM.

#### 3.5.2 Detection and identification of differential metabolites

Differential metabolite screening was conducted using VIP values, which reflect the importance of each variable in the first principal component of OPLS-DA. A higher VIP value indicates a greater contribution of the variable to the grouping. Additionally, a *t*-test was conducted to further evaluate the significance of metabolites between groups. Metabolites with VIP values greater than 1 and *P*-values less than 0.05 were considered as potential biomarkers. The analysis identified several potential biomarkers in both the RKM and SKM groups, including hydrocarbons, amino acids and their derivatives, organic acids, organic bases, aldehydes, esters, and fatty acids. Specifically, the expression of 21 metabolites was up-regulated, including acetylcholine chloride, niacinamide, pyridoxine, methyl jasmonate, dTMP, and cis-4-hydroxy-L-proline. Conversely, the expression of 136 metabolites was down-regulated, including L-aspartic acid, citric acid, guanosine monophosphate (GMP), L-lysine, L-methionine, and L-phenylalanine.

**Fig. 4:**
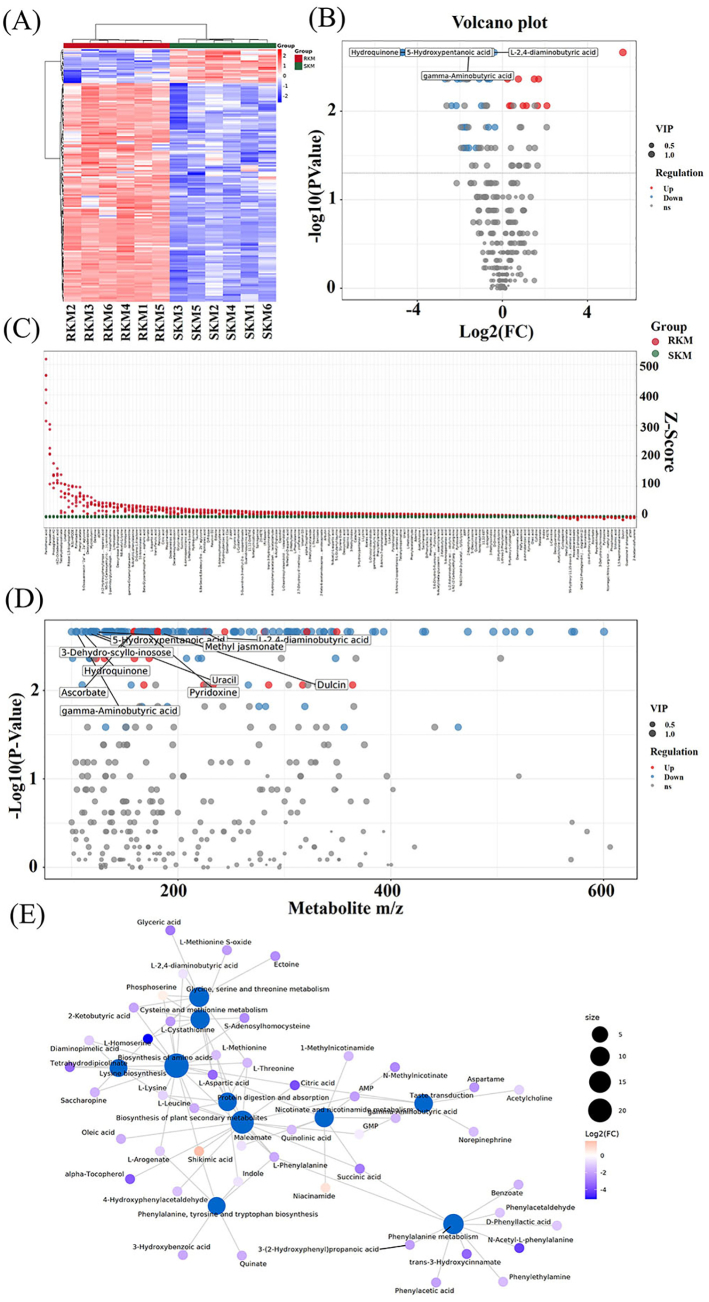
Detection and identification of differential metabolites. (A) Hierarchical clustering heatmap of differential metabolites. (B) Volcano Plot. (C) *Z*-score Plot. (D) Differential metabolic matter charge ratio and *P*-Value Scattering Plot. (E) Analysis of differential metabolite formation pathways.

Hierarchical clustering analysis (HCA) is a widely used method in data mining and statistics to explore the diversity in metabolite profiles across different experimental samples. In this study, HCA was employed to reveal variations among the metabolite profiles of the samples. As shown in Fig. 4A, the color gradient from blue to red represents the range of metabolite expression abundance, with blue indicating low expression and red indicating high expression. The color gradient clearly illustrates the differences in metabolite levels between RKM and SKM samples. Notably, the expression of differential metabolites was significantly down-regulated in steamed Antarctic krill, consistent with the previously observed results.

The standard score (*Z*-score) map provides a visual comparison of the relative contents of differential metabolites between the RKM and SKM samples at the same level. From the differential metabolite volcano plot (Fig. 4B), it is evident that the expression fold difference between the RKM and SKM samples is most pronounced. The four metabolites showing the most significant differential expression are hydroquinone, 5-hydroxypentanoic acid, gamma-aminobutyric acid, and L-2,4-diaminobutyric acid. Fig. 4C presents the standard scores of differential metabolites in Antarctic krill samples before and after steaming. The higher the value on the ordinate, the greater the metabolite content. This visualization provides an intuitive comparison of the relative contents of 157 differential metabolites between the RKM and SKM groups, highlighting that thermal induction caused significant changes in the samples. Fig. 4D displays a scatter plot of the mass-to-charge ratio versus *P*-value for the metabolites, offering a clear and intuitive view of the distribution of key differential metabolites. Metabolites with significant differential expression (*P* < 0.05) include methyl jasmonate, 3-dehydro-scyllo-inosose, uracil, and pyridoxine.

#### 3.5.3 Analysis of differential metabolite formation pathways in steamed Antarctic krill

The related metabolic network (Fig. 4E) illustrated the correlation of differential metabolites in Antarctic krill before and after thermal induction, and explored possible metabolic pathways of these metabolites. Amino acids emerged as the most frequently involved metabolites, forming a prominent cluster that includes phenylalanine, glycine and serine, key differential metabolites affected by steaming. Additionally, several amino acids, such as aspartic acid, methionine, lysine and leucine, which played important roles in the flavor of Antarctic krill, were significantly downregulated after steaming.

The observed reduction in amino acid levels may result from their participation in the Maillard reaction, where they react with glucose or other carbohydrates during heat processing, leading to a decrease in their content. This aligned with the previous findings that these changes in amino acids not only directly influenced the flavor characteristics of Antarctic krill but also synergize with nucleotides such as GMP and AMP, further enhancing or modifying the umami profile (Zhong et al., 2021). The identification of various nucleotide derivatives underscores their importance in flavor formation. Previous research (Chen et al., 2021) had similarly demonstrated the role of GMP and other nucleotides in enhancing the umami taste of seafood. Therefore, the flavor changes in Antarctic krill during steaming were the result of a multifaceted interplay between regulated metabolic pathways.

The mechanisms underlying flavor formation in steamed Antarctic krill are highly complex and involve the interplay of lipid oxidation, amino acid metabolism, and nucleotide interactions, among other biochemical processes. Amino acid metabolism and nucleotide metabolism jointly serve as key contributors, with the reduction of certain amino acids and the formation of nucleotide derivatives significantly shaping the flavor profile. The oxidation and degradation of lipids serve as primary pathways for flavor development. During steaming, unsaturated fatty acids, such as oleic and linoleic acids, undergo oxidation due to their chemical susceptibility (double bonds), producing hydroperoxides. These hydroperoxides subsequently decompose into various flavor-active compounds, including ketones, aldehydes, and alcohols. For instance, nonanal is a key product derived from oleic acid oxidation, while 1-octen-3-ol, a crucial odor-active compound, originates from linoleic acid degradation. Saturated and monounsaturated fatty acids also contribute to flavor by forming linear aldehydes and hydrocarbons (Farvin et al., 2012; Matsui et al., 2003). These lipid oxidation products form the backbone of the characteristic aroma of steamed Antarctic krill. They are further enriched through interactions with Maillard reaction intermediates.

The interplay between lipid oxidation and the Maillard reaction plays a pivotal role in flavor complexity. Lipid oxidation products, such as aldehydes, can react with Maillard reaction intermediates to generate secondary volatile compounds, including furans and alcohols. These cross-reactions enhance the sensory attributes of steamed krill by adding depth to its roasted and savory flavor. Aromatic amino acids, such as phenylalanine, also participate in metabolic pathways linked to secondary metabolite biosynthesis. These processes contribute to the chemical diversity of flavor compounds formed during steaming.

In summary, the flavor formation in steamed Antarctic krill is a result of a multifaceted interplay between lipid oxidation, amino acid metabolism, and Maillard reactions. Lipid-derived volatiles, amino acid degradation products, and nucleotide interactions collectively shape the distinctive umami and aromatic profile of krill. This study advances the understanding of molecular mechanisms involved in seafood flavor development and offers valuable guidance for optimizing processing techniques in the food industry.

## 4 Conclusion

This study provided a detailed analysis of the volatile compounds, metabolites, and taste characteristics of Antarctic krill before and after steaming. HS-SPME-GC–MS identified various volatile compounds, including alcohols, aldehydes, and ketones, which contribute to the krill's flavor profile. Notably, the content of nonanal and 1-octen-3-ol significantly increased after steaming due to lipid degradation. Metabolite profiling revealed substantial shifts in metabolite content, with significant downregulation of L-aspartic acid, citric acid, GMP, L-lysine, L-methionine and L-phenylalanine during steaming. Hydroquinone, gamma-aminobutyric acid, and L-2,4-diaminobutyric acid were identified as the most significantly altered metabolites. Metabolic network further linked these shifts to reactions involving amino acids, nucleotide and other compounds during steaming. The results highlighted that steaming significantly affects both the chemical composition and flavor of Antarctic krill, offering valuable insights into the metabolic transformations during food processing.

## CRediT authorship contribution statement

**Tingting Yang:** Writing – original draft, Software, Methodology, Investigation, Data curation. **Yang Liu:** Software, Methodology. **Jing Bai:** Writing – review & editing, Methodology. **Yan Fan:** Writing – review & editing, Visualization. **Ye Chen:** Methodology, Investigation. **Ping Dong:** Writing – review & editing. **Xia Yang:** Writing – review & editing, Software. **Hu Hou:** Writing – review & editing, Funding acquisition.

## Declaration of competing interest

The authors declare that they have no known competing financial interests or personal relationships that could have appeared to influence the work reported in this paper.

## Data Availability

Data will be made available on request.
